# Associations between immune-suppressive and stimulating drugs and novel COVID-19—a systematic review of current evidence

**DOI:** 10.3332/ecancer.2020.1022

**Published:** 2020-03-27

**Authors:** Beth Russell, Charlotte Moss, Gincy George, Aida Santaolalla, Andrew Cope, Sophie Papa, Mieke Van Hemelrijck

**Affiliations:** 1 Translational Oncology and Urology Research, King’s College London, London, UK; 2 Guy’s and St. Thomas NHS Foundation Trust, London, UK; 3 Centre for Rheumatic Diseases, King’s College London, London, UK; 4 School of Cancer and Pharmaceutical Sciences, King’s College London, London, UK; * All authors contributed equally.; ** Both senior authors contributed equally.

**Keywords:** immune modulation, immune suppression, cancer, COVID-19, adverse events

## Abstract

**Background:**

Cancer and transplant patients with COVID-19 have a higher risk of developing severe and even fatal respiratory diseases, especially as they may be treated with immune-suppressive or immune-stimulating drugs. This review focuses on the effects of these drugs on host immunity against COVID-19.

**Methods:**

Using Ovid MEDLINE, we reviewed current evidence for immune-suppressing or -stimulating drugs: cytotoxic chemotherapy, low-dose steroids, tumour necrosis factorα (TNFα) blockers, interlukin-6 (IL-6) blockade, Janus kinase (JAK) inhibitors, IL-1 blockade, mycophenolate, tacrolimus, anti-CD20 and CTLA4-Ig.

**Results:**

89 studies were included. Cytotoxic chemotherapy has been shown to be a specific inhibitor for severe acute respiratory syndrome coronavirus in in vitro studies, but no specific studies exist as of yet for COVID-19. No conclusive evidence for or against the use of non-steroidal anti-inflammatory drugs (NSAIDs) in the treatment of COVID-19 patients is available, nor is there evidence indicating that TNFα blockade is harmful to patients in the context of COVID-19. COVID-19 has been observed to induce a pro-inflammatory cytokine generation and secretion of cytokines, such as IL-6, but there is no evidence of the beneficial impact of IL-6 inhibitors on the modulation of COVID-19. Although there are potential targets in the JAK-STAT pathway that can be manipulated in treatment for coronaviruses and it is evident that IL-1 is elevated in patients with a coronavirus, there is currently no evidence for a role of these drugs in treatment of COVID-19.

**Conclusion:**

The COVID-19 pandemic has led to challenging decision-making about treatment of critically unwell patients. Low-dose prednisolone and tacrolimus may have beneficial impacts on COVID-19. The mycophenolate mofetil picture is less clear, with conflicting data from pre-clinical studies. There is no definitive evidence that specific cytotoxic drugs, low-dose methotrexate for auto-immune disease, NSAIDs, JAK kinase inhibitors or anti-TNFα agents are contraindicated. There is clear evidence that IL-6 peak levels are associated with severity of pulmonary complications.

## Introduction

Since the outbreak of severe acute respiratory syndrome (SARS)-CoV-2 or COVID-19 coronavirus started in China in December 2019, there is increasing evidence that those with existing comorbidities, older age or a compromised immune system are at higher risk of developing severe and even fatal respiratory diseases [1]. Cancer and transplant patients are also considered to be in this risk group [2, 3], especially as they may be treated with immune-suppressive or immune-stimulating drugs.

The current review focuses on the effects of immune-suppressive or immune-stimulating drugs on the host immunity against COVID-19. Here, we report a short introduction to each drug, followed by a summary of the results from the identified studies for each relative drug.

We hypothesise that the drugs selected will likely be categorised into one of two groups: 1) those that may be harmful for patients and put them at risk of increased morbidity/mortality associated with COVID-19 infection and 2) those that may be used to treat the immunopathology associated with severe persistent viral infection. The clinical impact of this review is, thus, twofold. It aims to identify which drugs clinicians should be thinking about taking patients off to protect them from increased harm from COVID-19 and also which drugs could be potentially beneficial in the fight against the disease.

This review covers the information available today. As the COVID-19 pandemic progresses, there is an opportunity and responsibility to collect prospective data using established randomised controlled trial involving drugs of interest and cohort-based translational studies.

1. All cytotoxic chemotherapy
2. Low-dose steroids and non-steroidal anti-inflammatory drugs (NSAIDs)
3. Any tumour necrosis factor (TNF) blocker
4. interlukin-6 (IL-6) blockade
5. Janus kinase (JAK) inhibitors
6. IL-1 blockade
7. Mycophenolate
8. Tacrolimus
9. Anti-CD20
10. CTLA4-Ig

## Methods

A search was conducted using Ovid MEDLINE and a total of 89 studies were included in this review for 10 different types of immune-suppressing or immune-stimulating drug groups. Table 1 shows the search terms and the number of studies included in the review.

## Results

### All cytotoxic chemotherapy

Cytotoxic chemotherapy inhibits cell division through multiple mechanisms. It may have therapeutic activity as compounds against corona viral strains. Moreover, cancer patients who have undergone chemotherapy may be at increased risk of developing symptoms of SARS.

#### Results

The search yielded 24 results, of which 18 were included (Supplementary Table 1). Below are key case-reports of interest to highlight:

- Coronavirus was one of the most common viral pathogens identified in paediatric cancer patients undergoing chemotherapy, second to human rhinovirus [4]. Viral co-detection was frequent in patients with cancer and acute respiratory infections.
- A brain biopsy was HCoV-OC43-positive by metagenomic next-generation sequencing in the case of a 1-year-old child with pre-B acute lymphoblastic leukaemia [5].
- A case of a young woman with stage IIIA breast cancer from 1999 reported that diagnosis of coronavirus following treatment for cancer using a high-dose chemotherapy regimen and autologous bone marrow and stem cell transplantation. The electron microscopy revealed coronavirus pneumonia [6]. Coronavirus should be potentially considered in the differential diagnoses of respiratory failure in patients who have undergone high-dose chemotherapy and autologous bone marrow transplantation.

In addition, some further observations have been made in relation to specific treatments:

- Although HIV protease inhibitors such as lopinavir has been suggested as a low-micromolar inhibitor of MERS-CoV, the different mechanistic classes that HIV and coronavirus fall under meant that there was low affinity for coronavirus strains compared to HIV [7].
- The following compounds have been shown to be active *in vitro* against the SARS-CoV virus: TNFα-converting enzyme inhibitor (TAPI-2); IFN-α (B/D, mDEF201 by adenovirus 5 vector, CR3014 humanized monoclonal antibody (a neutralising antibody specific for SARS-CoV), recombinant IFN-α2b and type I IFN-β); Interferon inducers (Ampligen and polyinosinic–polycytidylic); therapeutic antibodies (2978/10, equine anti-SARS-CoV F[ab’] and monoclonal antibody 201); attachment inhibitors (Urtica Dioica lectin and griffithsin); host immune system [8].
- 6-mercaptopurine (6MP) and 6-thioguanine (6TG) have been used in cancer chemotherapy for treatment of acute lymphoblastic or myeloblastic leukaemia and were found to be specific inhibitors for the SARS coronavirus [9].
- Carbohydrate-binding agents (CBA) may be able to block enveloped viruses other than HIV in their entry process and coronaviruses and influenza viruses are other examples of enveloped viruses that may be highly susceptible to the antiviral action of CBAs [10].
- The genome of SARS-CoV encodes five major proteins: the spike protein (S), the envelope protein (E), the membrane glycoprotein (M), and the nucleocapsid protein (N). M and E may help host cells to induce the production of protective IFN-α to fight against the virus. Bananin 1-[3-hydroxy-5-(hydroxymethyl)-2-methyl-4-pyridinyl]-2,8,9-trioxaadamantane-3,5,7-triol acts as zinc (Zn^2+^) chelator and is therefore of interest to target and inhibit immunodeficiency virus type 1 (HIV-1) zinc finger HIV-1 RNA-binding nucleocapsid protein p7 (NCp7). Bananin is converted to bananin 5’-monophosphate (BNP) which together with B6RA (vitamin A-vitamin B6 conjugate) and could inhibit infectious virion encapsidation. Targets of BNP and B6RA has shown to be present also in SARS-associated coronavirus making them possible therapeutic candidates [11].

#### Conclusion

Coronavirus strains were one of the most common viral pathogens identified in paediatric cancer patients undergoing chemotherapy. Patients with pre-B acute lymphoblastic leukaemia and breast cancer who have undergone chemotherapy have reported cases of coronavirus infection. Cytotoxic therapies used in cancer chemotherapy such as 6MP and 6TG have shown to be specific inhibitors for SARS coronavirus in *in vitro* studies. However, further *in vitro* and *in vivo* studies are required to confirm this, especially in COVID-19. Currently, there is no scientific evidence of the interaction between methotrexate and COVID-19.

### Low-dose steroids and NSAIDs

Since the outbreak of the novel COVID-19 infection, various contradictory information has been circulated regarding the potentially negative effect of treating patients with NSAIDs, non-NSAIDs and corticosteroids. NSAIDs work through inhibition of the cyclooxygenase enzymes (COX-1/COX-2), which are involved in the synthesis of key biological mediators. These mediators in turn control inflammation. Corticosteroids are involved in a number of key physiological processes including the immune response and inflammation and low-dose steroids are often prescribed to cancer patients with suppressed immune systems to prevent the development of related auto-immune diseases.

#### Results

A total of 58 studies were identified from the search terms, of which 13 were deemed suitable for inclusion (Supplementary Table 2). Our search did not identify any strong evidence for or against the use of ibuprofen for treatment of COVID-19 specifically. One study did however link SARS-CoV to the downregulation of angiotensin converting enzyme-2 (ACE2) which is upregulated by ibuprofen [12]. The authors of this study were investigating the link between the severity of COVID-19 symptoms in patients with asthma and hypertension.

The only other study to investigate a non-steroidal anti-inflammatory drug was one which looked at indomethacin [13]. This study suggested that indomethacin exhibited potent antiviral activity against canine coronavirus (dramatically inhibiting virus replication and protecting the host cell from virus-induced damage). This activity was also observed against human SARS-CoV at a concentration dose of 1 mg/kg.

In general, there appeared to be a few positive results for the use of corticosteroids in viral infections such as SARS-CoV [14-19]. Corticosteroids were widely used during the SARS-CoV outbreak due to their known ability to modulate a variety of involved cytokines (including IL-1, IL-6, IL-8, IL-12 and TNFα) [14, 16]. Various studies in humans noted that corticosteroids appeared effective in reducing immunopathological damage but concerns centred around the promotion of viral rebound and association with adverse events (including acute respiratory distress syndrome) [14]. One laboratory study which treated porcine respiratory coronavirus infected pigs with dexamethasone suggested that one or two doses of the corticosteroid in the acute phase of infection may effectively alleviate early pro-inflammatory response, but prolonged administration may play a role in enhancing viral replication [18]. A separate Chinese study, which separated SARS-CoV patients into 4 treatment groups, identified early high-dose steroids in combination with a quinolone as producing the best patient outcomes [19]. Nevertheless, one review stated that the WHO does not currently recommend corticosteroids in other viral diseases such as Dengue as the ‘glucocorticoid-mediated stimulation of the hypothalamic-pituitaryadrenal axis can also drive lymphocytopenia, or it may promote exaggerated pro-inflammatory responses that eventually cause a worsening of the pathogenic condition’ [20].

#### Conclusion

The current literature does not give conclusive evidence for or against the use of NSAIDs in the treatment of COVID-19 patients, though there appears to be some evidence that corticosteroids may be beneficial in the treatment of SARS-CoV. However, it is important to note this is not specific to COVID-19.

### TNFa blocker

TNF family of receptors and cytokines is very large and are often the targets for drugs. One example are TNFa inhibitors, which act by supressing the physiologic response to TNFa. TNFa is a pro-inflammatory cytokine involved in autoimmune and immune-mediated disorders such as rheumatoid arthritis, ankylosing spondylitis, inflammatory bowel disease, psoriasis, hidradenitis suppurativa and refractory asthma. Inhibitors of TNFa may be used in their treatment.

Inhibition of TNFa can be achieved with a monoclonal antibody, such as infliximab (Remicade), adalimumab (Humira), certolizumab pegol (Cimzia) and golimumab (Simponi), or with the receptor fusion protein etanercept (Enbrel).

#### Results

A total of three studies were identified, of which two were deemed suitable for inclusion (Supplementary Table 3). The first study was a research letter that suggested that TNFa has been implicated in the severe immune-based pulmonary injury caused by SARS coronavirus, suggesting that TNFa inhibitors could be a potential treatment for the acute respiratory disease syndrome caused by coronavirus [21]

The second study utilised 22 piglets to assess the efficacy of an anti-TNFa)therapy for endotoxin respiratory diseases and observed that TNFa blockade was not associated with decrease in disease severity [22].

#### Conclusion

Currently, there is no evidence indicating that TNFa blockade is harmful to patients in the context of COVID-19.

### IL-6 blockade

IL-6, promptly and transiently produced in response to infections and tissue injuries, contributes to host defence through the stimulation of acute phase responses, hematopoiesis, and immune reactions. Although its expression is strictly controlled by transcriptional and posttranscriptional mechanisms, dysregulated continual synthesis of IL-6 plays a pathological effect on chronic inflammation and autoimmunity [23]. For this reason, tocilizumab, a humanized anti-IL-6 receptor antibody was developed. Other approved anti IL-6 drugs are siltuximab (Sylvant) and sarilumab. Several agents are in clinical trials:olokizumab (CDP6038), elsilimomab, BMS945429(ALD518), sirukumab (CNTO 136), CPSI-2364, ALX-0061, clazakizumab, olokizumab, sarilumab, sirukumab, ARGX-109, FE301 and FM101^.^

#### Results

A total of 108 studies were identified from the second search strategy, 23 were suitable for inclusion (Supplementary Table 4). The first search strategy found no hits.

#### IL6 an actor in the pathogenetic mechanisms of the coronavirus infection

- COVID-19 induces a pro-inflammatory generation and secretion of cytokines including IL-1b and IL-6 via the toll like receptors (TLR) that causes the production of active mature IL-1b which is a mediator of lung inflammation, fever and fibrosis. Anti-inflammatory cytokines, such as IL1-Ra, IL-37 or IL-38 could potentially provide relief in both systemic inflammation and fever occurring after infection [24].
- Cytokine profiles in patients diagnosed with SARS showed marked elevation of T-helper lymphocyte type 1 (Th1) cytokine interferon-gamma (IFN-γ), inflammatory cytokines IL-1β, IL-6 and IL-12 for at least two weeks after disease onset. Children however presented a much milder cytokine and chemokine storm [16].
- The high levels of IL-6 in the acute stage associated with lung lesions found in SARS patients are activated by the viral nucleocapsid SARS-CoV N protein [25].
- Over induction of inflammatory cytokine and dysregulation of cytokine signalling has been observed in patients with SARS in comparison with other respiratory viruses including respiratory syncytial virus (RSV), influenza A virus (FluAV), and human parainfluenza virus type 2 (hPIV2). SARS-CoV and RSV induced high levels of IL-6 and RANTES compared with FluAV and hPIV2 [26].
- The N-protein of SARS-CoV induces pulmonary inflammatory reaction and acute lung injury, which were related to the increase and imbalance of pro-inflammatory and anti-inflammatory cytokines. Glucocorticoids could effectively alleviate the pulmonary inflammatory reaction induced by N-protein of SARS-CoV [15].
- SARS-CoV does not productively infect human macrophages (Mphi) or dentritic cells (DCs), however it modulates a massive release of IL-6 and IL-12 and compromises the endocytic capacity (e.g., antigen capture capture) of Mphi was significantly compromised [27].
- Changes in plasma T helper (Th) cell cytokines, inflammatory cytokines and chemokines in 20 patients diagnosed with SARS were assessed. The elevation of Th1 cytokine IFN-γ, inflammatory cytokines IL-1, IL-6 and IL-12 and chemokines IL-8, MCP-1 and IP-10 confirmed the activation of Th1 cell-mediated immunity and hyper-innate inflammatory response in SARS through the accumulation of monocytes/macrophages and neutrophils [17].

#### IL-6 as a potential marker of disease severity in coronavirus infected patients

- IL-6 blood measurements seem useful to diagnose severe COVID-19 cases. The findings suggest that IL-6 and D-Dimer level can be used to estimate the severity of COVID-19. The optimum critical point of IL-6 in the group was 24.3 pg/ml, which was the upper limit of no severe pneumonia [28].
- The increased expression of IL-2R and IL-6 in serum is expected to predict the severity of the 2019-nCoV pneumonia and the prognosis of patients [29].
- The serum levels of IL-6 and CXCL-10 were significantly elevated in MERS-CoV patients who developed severe diseases [30].
- A new lethal animal model was characterised for SARS-CoV. Strain v2163 had nine mutations that increased levels of IL-1alpha and IL-6 in mice. The high IL-6 expression was correlated with mortality [31].
- SARS vaccination was tested in a murine SARS model. A high level of IL-6 and on days 2 and 3 after SARS-CoV infection was closely linked to the virus replication and disease severity [32]
- Interleukin-6 (IL-6) and IL-8 are key SARS-CoV-induced epithelial cytokines capable of inhibiting the T-cell-priming ability of dendritic cells, a cellular element of the host innate defenses against respiratory infections, leading to an exacerbated inflammatory cascades and severe tissue damage in SARS patients [33].
- In patients with a diagnosis of SARS-associated coronavirus infection, there were no significant differences in peak levels of IL-6, IL-8 and TNF**α** between patients with and without acute respiratory distress syndrome. However , CRP and TNFα were associated with worse outcomes and might be used as prognostic markers of SARS [34].
- IFN-gamma, IL-18, TGF-beta, IL-6, IP-10, MCP-1, MIG were highly elevated in the acute phase sera of Taiwan SARS patients, being IL-18, IP-10, MIG, and MCP-1 were significantly higher in the death group than in the survival group. It suggests that an interferon-gamma-related cytokine storm was induced post SARS coronavirus infection [35].
- Eight patients with SARS [36] were treated with ribavirin, which was not effective in reducing the SARS coronavirus load in three of eight. Elevated levels of interleukin (IL)-6 and IL-8 subsequent to the peak viral load were found in eight and six cases [37].
- A cytokine profiling was performed for 110 serum from healthy donors, patients with SARS, patients with severe SARS, and patients with SARS in convalescence. IL6 concentrations were significantly elevated in severe SARS patients, but the IL-6 concentrations were similar in convalescent patients and control subjects which suggested that IL6 is associated with SARS severity [38].
- The authors of this study set out to study the inflammatory cytokine profile in children with SARS. They found that the plasma concentrations’ key proinflammatory cytokines, including IL-6, were not substantially increased in any of the patients throughout the course of illness. From this, the authors stated that the cytokine results cast doubt on the liberal use of corticosteroids in paediatric SARS patients [39].

#### Potential targets for therapeutic intervention related to IL6 regulation during infection

- ADAM-17 regulates IL-6 class switching as a mediator between pro- and anti-inflammatory responses to viral antigenic stimuli in Ebola, SARS-CoV and dengue infections in humans. Therefore, ADAM-17 should be considered as a potential target molecule for novel antiviral drug discovery for infections, such as SARS-CoV [20]
- SARS-CoV ssRNA is a new therapeutic target given its capacity to cause acute lung injury in mice with a high mortality rate *in vivo* experiment suggesting that SARS-CoV specific GU-rich ssRNA plays a very important role in the cytokine storm associated with a dysregulation of the innate immunity [40].
- DUSP1 and p38 MAPK are potential therapeutic targets for coronavirus infectious bronchitis virus, given their capability to reduce the production of an excessive amount of IL-6 and IL-8 in the infected cells [41].

#### Potential therapeutic agents that inhibit the infection-induced production of IL6

- Pretreatment of primary cultures of human nasal and tracheal epithelial cells with glycopyrronium or formoterol decreased viral RNA levels and/or titers, the expression of the HCoV-229E and the infection-induced production of cytokines, including IL-6, IL-8 and IFN-beta. Treatment of the cells with the CD13 inhibitor 2’2’-dipyridyl decreased viral titers. Pretreatment of the cells with a combination of three drugs (glycopyrronium, formoterol and budesonide) exerted additive inhibitory effects on viral titers and cytokine production [42].

#### Conclusion

It has been suggested that the pathogenesis of SARS-CoV is mediated by disproportional immune responses and the ability of the virus to circumvent innate immunity. The COVID-19 infection has also been observed to induce pro-inflammatory cytokine generation and secretion of cytokines, such as IL-6, which dysregulates the local inflammatory responses that have been suggested as partially responsible for the devastating acute respiratory distress syndrome.

Moreover, it has been observed that COVID-19 induces high levels of IL-6 for at least 2 weeks after disease onset. Children presented lower levels of cytokine production. IL-6 has been suggested as a potential prognostic marker of COVID-19 disease severity. Different molecules related with the IL6 pathway have been suggested as potential therapeutic targets such as ADAM-17, SARS-CoV ssRNA, DUSP1 and p38 MAPK.

Currently, there is no scientific evidence of the beneficial impact of IL-6 inhibitors in the modulation of the COVID-19 infection. Further understanding of the role of IL-6 reduction will be forthcoming as the pandemic progresses and further clinical data becomes available. *In vitro* treatment with glycopyrronium, formoterol and budesonide exerted additive inhibitory effects on viral titers and cytokine production human nasal and tracheal epithelial cells.

### JAK inhibitors

JAK inhibitors work by inhibiting the activity of one or more of the JAK family of enzymes, including, JAK1, JAK2, JAK3 and TYK3. JAKs interact with signal transducer and activator of transcription proteins (STATs) and the JAK-STAT pathway is central to cellular response to exogenous signals in the immune system. The JAK family of enzymes are responsible for signal transduction and JAK inhibitors play a major role in inhibiting and blocking cytokine release that can contribute to growth of malignant cells in cancer. JAK inhibitors are used in the treatment of cancer and inflammatory diseases such as rheumatoid arthritis [43].

This review focuses on how the JAK-STAT pathway can be manipulated to reduce viral entry and inflammation in patients with coronavirus. The main targets that the review highlighted were baricitinib (JAK inhibitor), IRE1α (an endoplasmic reticulum stress sensor, leading to an increased expression of negative regulators of JAK-STAT SOCS1 and SOCS3) and combination therapies using tylophorine-based compounds with JAK2 inhibitors.

#### Results

Fifteen studies were yielded from the search of which 4 were deemed suitable for inclusion (Supplementary Table 5).

#### Baricitinib

Baricitinib (Olumiant) is a JAK inhibitor that was approved by the European Medicines Agency in February 2017 for the treatment of moderate-to-severe active rheumatoid arthritis in adults with an inadequate response to one or more disease-modifying anti-rheumatic drugs [44]. One study suggested the use of Baricitinib to reduce the viral entry and inflammation caused by 2019-nCoV. Most viruses enter cells through receptor-mediated endocytosis. ACE2, a cell-surface protein expressed on cells in the kidney, blood vessels, heart, and alveola type 2 (AT2) cells in the pulmonary epithelia, may be the receptor that 2019-nCoV uses to infect lung cells. The authors of Richardson *et al* [45] suggest that by inhibiting adaptor associated protein kinase 1 (AAK1) receptor that promotes endocytosis involved in ACE2, Baricitinib may reduce both the viral entry and the inflammation in 2019-nCoV patients.

#### IRE1α

Inositol-requiring transmembrane kinase/endoribonuclease 1α (IRE1α) is an endoplasmic reticulum stress sensor that leads to increased expression of negative regulators of JAK-STAT, suppressor of cytokine signalling (SOCS)-1 and SOCS-3 [46]. Therefore, IRE1α may be a novel target against coronavirus infection requiring further exploration.

#### Tylophorine-based compounds

Tylophorine-based compounds are isolated from plants and exert potent anti-coronaviral activities against SARS-CoV and MERS-CoV [47]. Nuclear factor kappa-light-chain-enhancer of activated B cells (NF-κB) activation is a common pro-inflammatory response of host cells to viral infection. Following *in vitro* analysis, Yang *et al* [48] suggests the use of a combination therapy for SARS-CoV or MERS-CoV, wherein a tylophorine compound known to target transmissible gastroenteritis virus and a JAK2 inhibitor synergise to block the alternative dominant NF-κB activation mediated by JAK2. Therefore, the combination treatment for the inhibition of coronavirus per se, e.g. viral genome replication, and blocking cellular NF-κB activation by coronaviruses, is a promising approach for the development of anti-coronavirals.

#### Conclusion

Current studies suggest that although there are potential targets in the JAK-STAT pathway that can be manipulated in the treatment for coronaviruses, they are all in early stages and require further *in vitro* and *in vivo* studies to confirm their therapeutic effects.

### IL-1 blockade

IL-1 is a pro-inflammatory cytokine and an important mediator of local and systemic inflammation. Excessive IL-1 release during viral infections can cause lung and tissue inflammation, fever and fibrosis. IL-1 suppression has found to be effective in many inflammatory diseases including rheumatoid arthritis [24].

It is well established that an over-expression of interleukin-1 is a hallmark of SARS-CoV infection, probably through activation of transcription factor nuclear factor, activator protein 1 and activating factor 2. In COVID-19 specifically, the virus is thought to bind to TLRs which activate the formation of pro-IL-1 and activation of the inflammasome [24]. This inflammasome activation is important for the regulation of cells of both the innate and adaptive immune system paving the way for specific immune responses. As part of the inflammasome activation, IL1-b is subsequently produced which mediates the inflammation of the lungs, fever and fibrosis thus causing respiratory complications in the infected host.

#### Results

A total of 37 studies were identified from the search of which 9 were deemed suitable for inclusion (Supplementary Table 6). Many of the studies were *in vitro* studies and repeatedly demonstrated increased IL-1 levels in patients infected with a coronavirus. One study investigated the levels of various inflammatory cytokines in 29, COVID-19 patients in China and compared the levels between general, severe and critically ill groups [49]. The authors reported no significant differences in IL-1b levels between the three groups of patients. Another study involving 20 consecutive SARS patients admitted to a Hong Kong hospital identified significantly elevated levels of IL-1β within the first 12, 7 and 5 days following onset of infection [17]. Those patients with more severe disease were treated with pulsed methylprednisolone and IL-1β levels returned to normal after 7 days. The seven patients with less severe disease did not receive any dosage of corticosteroids and their cytokine levels returned to normal range levels over the same 7-day time period.

A further Chinese study also identified reduced levels of IL-1β following administration of corticosteroids- suggesting inhibition of pro-inflammatory cytokines such as IL-1 may be a beneficial treatment strategy for treatment of SARS [50]. A third study, which measured serum cytokine levels in four patient groups including controls, patients with SARS, patients with severe SARS and convalescent SARS patients suggested that longer term treatment (over a period of 7-10 days) with low-dose steroids can alter serum cytokine levels, including IL-1α [38].

One rat model showed promising results for an IL-1 receptor antagonist which reduced the chemokine expression in infected animals [51]. However, this result cannot be generalised for humans. Unfortunately, one study, in which the authors state the ‘demonstrate for the first time that inflammation by coronavirus may be inhibited by anti-inflammatory cytokines belonging to the IL-1 family’, was only available as an abstract [52]. Therefore, further evidence or information to back this claim up is not available.

#### Conclusion

Overall, this review demonstrated that although it is evident that IL-1 is elevated in patients infected with a coronavirus, there is not at present evidence for an established role for IL-1 blockers in the treatment of COVID-19 in humans. The literature did, however, suggest a potential role for low-dose corticosteroids to reduce levels of pro-inflammatory markers, such as IL-1, which are elevated as part of the immune response and may have a role in the severe lung damage associated with human coronaviruses.

### Mycophenolate

Mycophenolate mofetil (which is a derivative of mycophenolic acid (MPA)) is an immune suppressant, antineoplastic and antiviral mediation. According to the British National Formulary, mycophenolate mofetil is used for the prophylaxis of acute rejection in renal transplantation and is usually used in combination with a corticosteroid and ciclosporin.

#### Results

Almost all of the studies investigated MPA as a potential therapy for MERS-CoV due to its anti-viral properties. Six of the 13 selected studies were *in vitro* studies, two were *in vivo,* one was a clinical example and four were reviews (therefore there was some overlapping of results) (Supplementary Table 7).

#### In-vitro studies

In general, the *in vitro* studies looked positive with MPA targeting the papain like proteases of both MERS-CoV and SARS-CoV [53, 54]. The studies found that MPA showed strong inhibition of the virus with a very low IC50 [55-57].

#### In vivo study

MPA can been used in combination with interferon-beta (IFN-b). One study, which applied this regime in marmosets exhibiting a severe disease resembling human MERS, reported high viral loads with more severe or even fatal disease [28]. The authors of this study state that MPA is likely to cause more harm than benefit to MERS patients.

#### Clinical studies

According to the review by Mo and Fisher [58], MPA monotherapy had not been tested in a clinical setting for the treatment of MERS-CoV. Al Ghamdi *et al* [59], presented an example where eight patients were treated with MPA for MERS-CoV, seven in combination with IFN-β. All eight of these patients survived, however the review by Mo and Fisher stated that this group of patients had lower Acute Physiology and Chronic Health Evaluation II scores compared with others in the cohort who received a variety of antiviral agents including ribavirin and IFN-a, steroids and antibiotics. Therefore, the results must be interpreted with caution.

#### Conclusion

Whilst the *in vitro* studies showed promising results for MPA against MERS, the *in vivo* studies suggest that its use is likely to cause more harm than benefit and hence is not likely to be useful against coronavirus infections. The clinical studies are too small to confirm or deny any beneficial use for MERS-CoV patients.

### Tacrolimus

Tacroliumus, also known as fujimycin, envarsus or FK506, is an immunosuppressive drug which is mainly administered after allogeneic organ transplant to lower risk of organ rejection. It’s mechanism of action focusses on inhibition of calcineurin which is involved in the production of IL-2. IL-2 is a cytokine which promotes the development and proliferation of T cells which form a vital component of the human adaptive immune response.

#### Results

A total of 18 studies were identified from the search terms, of which three were deemed suitable for inclusion (Supplementary Table 8).

Overall, the literature appeared to suggest a potential role for tacrolimus in the treatment of human coronaviruses. In a case study of two renal transplant recipients who tested positive for MERS CoV, a patient who was being treated with an immunosuppressive regimen of tacrolimus underwent full recovery whilst the other patient (who was not on this treatment regimen) succumbed to the infection [60]. The patient who eventually made a full recovery was also treated with antibacterial therapy and a reduced dose of mycophenolate mofetil and it is therefore not possible to conclude that patient recovery due to tacrolimus.

The two other included studies were both laboratory studies involving cell line culture. The first investigated pathways of coronavirus viral replication as potential antiviral therapeutic targets [61]. Genome-wide SARS-CoV yeast-two-hybrid interaction screen with human cDNA libraries identified FK506-binding proteins as interaction partners of SARS-CoV non-structural protein 1. Subsequently, the authors investigated whether tacrolimus inhibits viral replication of human coronaviruses. VeroFM cells infected with SARS-CoV and other human coronaviruses were treated with FK506. Results showed that FK506 effectively inhibited viral replication of SARS-CoV, HCoV-NL63 and HCoV-229E at non-toxic, low-micromolar concentrations with a reduction in viral titers to undetectable levels. The second study further confirmed this inhibition using novel non-immunosuppressive derivatives of FK506 in the context of HCoV-NL63 at low-micromolar, non-cytotoxic concentrations in cell culture [62].

#### Conclusion

Overall the small amount of literature available suggests a potential role of FK506 (tacrolimus) as a potent antiviral in the treatment of human coronaviruses. It is important to note, however, that COVID-19 is a novel disease and may have different aetiology and mechanistic action compared to existing strains and to date, this immunosuppressive drug and its derivatives has not been tested in humans. Further study is warranted, both in the clinical setting and laboratory.

### Anti-CD20

No studies were identified for inclusion.

### CTLA-4 Ig

No studies were identified for inclusion.

## Overall conclusion

The rapidly progressing SARS-CoV-2 pandemic has led to challenging decision-making about the treatment of critically unwell patients with the novel viral infection. In parallel, doctors across multiple specialties are making clinical decisions about the appropriate continuation of treatments for patients with chronic illnesses requiring immune-suppressive medication. This systematic review looks to provide guidance from the current available literature.

As the COVID-19 pandemic progresses, collective effort to capture data from prospective trials is required. Sponsors of randomized controlled trials recruiting patients randomized to receive immune modulatory drugs that may be affected by COVID-19 should collect data about the disease outcomes and consider interim analysis of potential advantages and disadvantages associated with using one of these medications.

Low-dose prednisolone and tacrolimus therapy may have beneficial impacts on the course of SARS-CoV-2. This observation requires further validation. The mycophenolate mofetil picture is less clear, with conflicting data from pre-clinical studies. There is no definitive evidence that specific cytotoxic drugs, low-dose methotrexate for auto-immune disease, NSAIDs, JAK kinase inhibitors or anti-TNFa biological agents are contraindicated. There is evidence that IL-6 peak levels are associated with severity of pulmonary complications. Ongoing studies of blockade of the IL-6 pathway are rational and will hopefully inform practice as the pandemic progresses.

## Funding declaration

We would like to thank our various funders: Guy’s and St Thomas’ Charity, Cancer Research UK (C45074/A26553), and the CRUK King’s Health Partner Centre.

## Conflicts of interest

The authors have no conflicts of interest to declare.

## Figures and Tables

**Supplementary Table 1.** Current studies investigating COVID-19 and cytotoxic chemotherapy.

Study Ref	Title	Authors	Country of study	Year	Summary of results
[1]	The differential diagnosis of pulmonary infiltrates in cancer patients during the outbreak of the 2019 novel coronavirus disease	Zhu *et al*	China	2020	COVID-19 complicates the clinical scenario of pulmonary infiltrates in cancer patients. Active treatment against the infection and patient surveillance should be initiated if infectious disease is considered.
[2]	Chemotherapy strategy for colorectal cancer under the outbreak of novel coronavirus pneumonia	Li *et al*	China	2020	Recommendations based on stage of cancer: Recurrent metastatic colorectal cancer: low-intensity maintenance therapy Patients with tumour changes or higher malignancy still need to receive combined chemotherapy. After radical surgery, given the relatively limited benefits of adjuvant chemotherapy, the intensity and duration of treatment can be reduced. In the face of patients with febrile tumour chemotherapy, it is necessary to analyse the cause of the fever of the patient. Stable disease and good general condition: elect to delay the time of imaging evaluation.
[3]	Fatal encephalitis associated with coronavirus OC43 in an immunocompromised child	Nilsson *et al*	Sweden	2020	A one-year-old child with pre-B acute lymphoblastic leukaemia (ALL) developed fatal encephalitis associated with human coronavirus OC43 (HCoV-OC43). During chemotherapy the child had a persistent HCoV-OC43 respiratory infection and later developed progressive encephalitis. Cerebrospinal fluid was negative for pathogens including HCoV-OC43, but a brain biopsy was HCoV-OC43-positive.
[4]	Recent developments in anti-severe acute respiratory syndrome coronavirus chemotherapy	Barnard and Kumaki	England	2011	Anti-SARS-CoV therapies recently published from 2007 to 2010 reviewed in this paper and the following compounds have been shown to be active in vitro against the virus: TNF-α-converting enzyme inhibitor (TAPI-2); IFN-α (B/D, mDEF201 by adenovirus 5 vector, CR3014 humanised monoclonal antibody, recombinant IFN-α2b and type I IFN-β); Interferon inducers (Ampligen and polyinosinic–polycytidylic); therapeutic antibodies (2978/10, equine anti-SARS-CoV F[ab’] and monoclonal antibody 201); attachment inhibitors (Urtica Dioica lectin and griffithsin); host immune system.
[5]	A 3-Year Retrospective Study of the Epidemiology of Acute Respiratory Viral Infections in Pediatric Patients With Cancer Undergoing Chemotherapy	Aydin Köker et al	Turkey	2019	Nasopharyngeal aspirates were analysed in patients younger than 21 years with acute respiratory infections. Coinfection with 2 viruses was present in 20.5% (45/219) of the episodes. Most frequent coinfections of respiratory viruses: 6 cases of HRV+hBoV, 5 of HRV, +AdRV, 4 of PI3+CoV 43, 3 of HRV+CoV 43, 3 of HRV, +IF A/H1N1, 3 of HRV+RSV A/B, and 2 of HRV+EV Detected more often in the months of January (16%) and October (15%) than in the other months Conclusion: no increase in mortality of cancer patients but cause for significant delays to chemotherapy, which might have an indirect impact on patient survival rates.
[6]	Frequent Respiratory Viral Infections in Children with Febrile Neutropenia - A Prospective Follow-Up Study	Söderman *et al*	Sweden	2016	Nasopharyngeal aspirates were collected during 87 episodes of febrile neutropenia in children age 0-18 years No symptoms were apparent in four episodes involving RV and one episode involving HCoV Persistent HCoV with a median follow-up time of 31 days High viral loads were correlated to more symptoms Respiratory viruses play an etiologic role in febrile neutropenia in children receiving treatment for a malignancy.
[7]	An Overview of Severe Acute Respiratory Syndrome-Coronavirus (SARS-CoV) 3CL Protease Inhibitors: Peptidomimetics and Small Molecule Chemotherapy	Pillaiyar *et al*	USA	2016	The paper focuses on on the status of various efficacious anti-SARS-CoV 3CLpro chemotherapies discovered during the last 12 years (2003−2015) from all sources, including laboratory synthetic methods, natural products, and virtual screening. Conclusion: No coronavirus protease inhibitor has yet successfully completed a preclinical development program
[8]	Respiratory Viral Infections in Patients With Cancer or Undergoing Hematopoietic Cell Transplant. [Review]	Hijano *et al*	Switzerland	2018	Clinical manifestations vary significantly depending on the type of virus and the type and degree of immunosuppression. Risk factors associated with prolonged viral shedding can include viral load, use of steroids, and myeloablative conditioning. Mortality associated with coronavirus in HCT recipients was inconclusive. While some studies [9] reported no associations, other studies [10] reported similar mortality rates in HCT recipients to those observed with other viruses such as RSV, influenza virus, and PIV.
[11]	Progress in Anti-SARS Coronavirus Chemistry, Biology and Chemotherapy	Ghosh et al	USA	2007	SARS-CoV proteases are attractive targets for the development of antiviral drugs to reduce viral replication and pathogenicity. Glycyrrhizin showed inhibitory activity for SARS-CoV replication but it has high cytotoxicity. HIV protease inhibitor nelfinavir, antihelminthic drug niclosamide and antimalarial agent chloroquine have also showed strong inhibitory activity against SARS-CoV replication. A human lgG1 form of 80R was found to bind the S1 domain of the SARS-CoV S protein (with a higher affinity comparable to that of ACE2 suggesting that the 80R human monoclonal antibody is a useful viral entry inhibitor for SARS treatment.
[12]	Alternative screening approaches for discovery of Middle East respiratory syndrome coronavirus inhibitors	LaFemina, RL.	USA	2014	Lopinavir is an HIV protease inhibitor. Lopinavir has been suggested as a low-micromolar inhibitor of MERS-CoV. However, proteases of HIV and coronaviruses fall into different mechanistic classes of proteases.
[13]	Acute respiratory viral infections in paediatric cancer patients undergoing chemotherapy	Benites *et al*	Brazil	2014	Paediatric patients with cancer and acute respiratory infection [14] and/or fever. Coronavirus was identified in 6.8% in the 50 samples of respiratory cases. Human rhinovirus (HRV) was the most common viral pathogen, followed by coronavirus, respiratory syncytial virus (RSV), and metapneumovirus, demonstrating the importance of these pathogens in the studied population. The prevalence of respiratory viruses was relevant in the infectious episode, with no increase in morbidity and mortality. Viral co-detection was frequent in patients with cancer and ARIs. The link of whether severe acute infection was directly related to the type of cancer or viral pathogen was not identified in the study.
[15]	Thiopurine analogue inhibitors of severe acute respiratory syndrome-coronavirus papain-like protease, a deubiquitinating and deISGylating enzyme	Chen *et al*	Taiwan	2009	6-mercaptopurine (6MP) and 6-thioguanine (6TG) have been used in cancer chemotherapy for treatment of acute lymphoblastic or myeloblastic leukaemia and were found to be specific inhibitors for the SARS coronavirus.
[14]	Carbohydrate-binding agents: a potential future cornerstone for the chemotherapy of enveloped viruses?	Balzarini, J.	Belgium	2007	Carbohydrate-binding agents (CBA) may be able to block enveloped viruses other than HIV in their entry process and coronaviruses and influenza viruses are other examples of enveloped viruses that may be highly susceptible to the antiviral action of CBAs. In HIV, glycan deletions in gp120 delays the spread of virus. CBA administration may bring the viral infection under control before glycan deletions occur and the immune system can get involved in the further clearance of the virus.
[16]	Molecular biology investigation of respiratory viruses as a factor of infectious complications in hemoblastosis and myelodepression	Chebotkevich et al	Russia	2006	Only abstract available, article in Russian and not accessible. Communicable respiratory viruses were investigated in 51 patients as a causative factor of infectious complication in hemoblastosis and myelodepression Coronaviruses detected in 13.7%
[17]	Synthesis of novel test compounds for antiviral chemotherapy of severe acute respiratory syndrome [18]. [Review]	Kesel AJ	United Arab Emirates	2005	25 test materials including interferon-inducer Bananin (BN) was an effective inhibitor of SARS-CoV in cell culture.
[19]	Induction of Th1 type response by DNA vaccinations with N, M, and E genes against SARS-CoV in mice	Huali *et al*	China	2005	The M and E are play a role in coronaviral particle assembling. Targeting these agents may lead to immune responses by inducing the production of protective IFN-α. N, M, and E genes may be used as the targets to prevent SARS-CoV infection.
[20]	A system of protein target sequences for anti-RNA-viral chemotherapy by a vitamin B6-derived zinc-chelating trioxa-adamantane-triol.	Keseal AJ.	Germany	2003	Bananin acts as zinc (Zn2+) chelator Targets and inhibits zinc finger of HIV-1 RNA-binding nucleocapsid protein p7 (NCp7). Bananin is converted to bananin 5’-monophosphate (BNP) which together with B6RA (vitamin A-vitamin B6 conjugate) and could inhibit infectious virion encapsidation. Targets of BNP and B6RA have shown to be present also in SARS-associated coronavirus making them possible therapeutic candidates.
[21]	Coronavirus Pneumonia Following Autologous Bone Marrow Transplantation for Breast Cancer	Folz and Elkordy	USA	1999	Case report: coronavirus in woman with stage III breast cancer following treatment with hig-dose chemotherapy and autologous bone marrow and stem cell transplant.

**Supplementary Table 2.** Current studies investigating COVID-19/ other coronavirus strains and low-dose steroids.

Study Ref	Title	Authors	Country of study	Year	Summary of results
[22]	Understanding SARS-CoV-2-Mediated Inflammatory Responses: From Mechanisms to Potential Therapeutic Tools	Fu, Cheng and Wu	China	2020	This review suggests that: SARS-CoV S protein can downregulate ACE2 Loss of pulmonary ACE2 function has been suggested to be associated with acute lung injury; the reduction in ACE2 function can cause dysfunction of the renin-angiotensin system [23] and enhance inflammation and vascular permeability. ACE2-associated lung injury has been suggested in SARS- CoV infection From reading this article the following article was then discovered…
[24]	Are patients with hypertension and diabetes mellitus at increased risk for COVID-19 infection?	Fang, Karakiulakis and Roth	Greece	2020	The expression of ACE2 is substantially increased in patients with type 1 or type 2 diabetes, who are treated with ACE inhibitors and angiotensin II type-I receptor blockers (ARBs). Hypertension is also treated with ACE inhibitors and ARBs, which results in an upregulation of ACE2 ACE2 can be increased by thiazolidinediones and ibuprofen. These data suggest that ACE2 expression is increased in diabetes and treatment with ACE inhibitors and ARBs increases ACE2 expression. Consequently, the increased expression of ACE2 would facilitate infection with COVID-19. The authors therefore hypothesise that diabetes and hypertension treatment with ACE2-stimulating drugs increases the risk of developing severe and fatal COVID-19.
[25]	Drug treatment options for the 2019-new coronavirus (2019- nCoV)	Lu	China	2020	No evidence given for or against using ibuprofen only that ‘anti-inflammatory drugs (such as hormones and other molecules)’ are potential therapeutic options for 2019-nCoV.
[26]	Severe acute respiratory syndrome: clinical and laboratory manifestations.	Lam, Chan, Wong	China	2004	Corticosteroid treatment reduced interleukin 8 (IL-8), monocyte chemoattractant protein-1 (MCP-1) and IFN-γ-inducible protein-10 (IP-10) concentrations from 5 - 8 days after treatment in SARS patients. These were all inflammatory markers which were remarkably increased in SARS patients. NB. This study is not specifically looking at SARS-CoV2 only at SARS-CoV.
[27]	Decoding the enigma of antiviral crisis: Does one target molecule regulate all?	Mahmud-Al-Rafat *et al*	Bangladesh, Germany, Canada, UK and Australia	2019	This review article aimed to look at multiple diseases caused by various viruses including Dengue, Ebola and SARS-CoV. They stated that ribavirin (an antiviral) is the most frequently used drug to combat SARS-CoV, and is administered together with corticosteroids. Although immunosuppressive corticosteroid drugs are commonly used in an attempt to reduce fatality rates, the results of clinical trials are not sufficiently satisfactory to approve as an effective class of therapeutic Glucocorticoid-mediated stimulation of the hypothalamic-pituitaryadrenal axis can also drive lymphocytopenia, or it may promote exaggerated pro-inflammatory responses that eventually cause a worsening of the pathogenic condition. (Please note this is not specific for SARS-CoV-19 and is referring to the 3 viral diseases mentioned).
[28]	The management of coronavirus infections with particular reference to SARS.	Wong SS, Yuen KY		2008	HCoV-OC43 and HCoV-229E initial principal pathogens discovered in 1960’s; acute respiratory diseases of less severity and mortality than SARS-CoV which promoted rapid search for effective antiviral treatments. Immunomodulatory agents (primarily corticosteroids) widely used during SARS to avoid excessive tissue damage by cytokine dysregulation but benefit of use not conclusively demonstrated. Multiple studies demonstrated increased plasma viral load with use of corticosteroids (RCT measured plasma viral load at regular intervals in non-intubated cases found higher concentrations of SARS-CoV RNA in week 2/3 of illness compared to patients who received placebo). 9.9% of patients in Hong Kong cohort study also suffered opportunitistic infections such as aspergillosis and late sequelae (including avascular osteonecrosis) with corticosteroid use.
[29]	Cytokine responses in porcine respiratory coronavirus-infected pigs treated with corticosteroids as a model for severe acute respiratory syndrome.	Zhang X, Alekseev K, Jung K, Vlasova A, Hadya N, Saif LJ	USA	2008	Laboratory study which treated porcine respiratory coronavirus infected pigs with corticosteroid dexamethasone as a model for SARS. Goal to define whether corticosteroid treatment altered immunity and lung inflammatory response at cytokine level and how changes correlated with pig lung lesions. Confirmed infection model applicable and resembled SARS. Treatment with dexamethasone suppressed early local IL-6 levels but increased later on in disease. Suggests may decrease inflammation at early infection stage but not later. Results also suggest that dexamethasone induced reduction of IL-6 might play a role in delayed onset of lung lesions but increases of IL-6 at later infection stage may contribute to severity of lung lesions (mimicking delayed onset of disease severity as seen in SARS patients treated with corticosteroids). **Overall findings suggest that 1 or 2 doses of dexamethasone in acute phase of infection may effectively alleviate early pro-inflammatory response, but prolonged DEX administration may play role in enhancing viral replication.**
[30]	Indomethacin has a potent antiviral activity against SARS coronavirus.	Amici C, Di Caro A, Ciucci A, Chiappa L, Castilletti C, Martella V, Decaro N, Buonavoglia C, Capobianchi MR, Santoro MG	Italy	2006	Utilised observation that cyclopentenone cyclooxygenase (COX) metabolites are active against several RNA viruses to investigate effect of COX inhibitor indomethacin on coronavirus replication. Indomethacin is common NSAID. Report describes how indomethacin was unexpectedly found to possess potent antiviral activity against canine coronavirus (dramatically inhibit virus replication and protect the host cell from virus-induced damage). Activity also observed in vivo and against human SARS-CoV. Oral administration of concentration 1mg/kg found to be effective.
[31]	A study of pulmonary inflammatory reaction induced by N-protein of SARS-CoV in rat models and effects of glucocorticoids on it	Hao D, He LX, Qu JM, Pan J, Hu BJ, Zhang J, Li ZZ	China (full article only available in Chinese but informative abstract)	2005	Studied pulmonary inflammatory reaction induced by N-protein of SARS-CoV in rat models and effects of glucocorticoids on inflammatory reaction. Mice treated with dexamethasone. N-protein of SARS-CoV shown to have pathogenicity inducing pulmonary inflammatory reaction and acute lung injury which were related to the increase and imbalance of pro-inflammatory and anti-inflammatory cytokines. Glucocorticoids (dexamethasone) demonstrated effectively alleviate pulmonary inflammatory reaction.
[32]	Overview of antiviral and anti-inflammatory treatment for severe acute respiratory syndrome.	Chihrin S, Loutfy MR	Canada	2014	Anti-inflammatory agents heavily utilised during SARS outbreak as it was believed that the development and worsening of the pulmonary disease had inflammatory aetiology and pathogenesis. Immunopathological analysis of 20 SARS patients demonstrated increased Th1 cytokine interferon y and inflammatory cytokines IL-1, IL-6 and IL-12 for > 2 weeks following onset of symptoms. Also observed decrease in viral activity and IgG seroconversion during 2nd week of illness despite general progression of respiratory disease- suggesting respiratory damage is secondary to immune-mediated inflammatory response. Corticosteroids used during SARS outbreak for their ability to module variety of involved cytokines. Numerous papers published outcomes of patients administered corticosteroids (mostly IV hydrocortisone or methylprednisolone) in either early low-dose or pulsed-dose strategies. Most concluded corticosteroids appeared to be effective in reducing immunopathological damage (resulting in improved and rapid resolution of symptoms). Opposition to corticosteroid use centred around use of corticosteroid use in treatment of acute respiratory distress syndrome- concern that corticosteroid treatment may weaken immune response and promote viral rebound. Reports also add evidence to theory. Further opposition includes association with adverse events such as hyperglycaemia, hypokalaemia, hypertension, gastrointestinal haemorrhage and avascular necrosis (AVN).
[33]	High-dose hydrocortisone reduces expression of the pro-inflammatory chemokines CXCL8 and CXCL10 in SARS coronavirus-infected intestinal cells.	Cinatl J Jr, Michaelis M, Morgenstern B, Doerr HW	Germany (needs to be purchased)	2005	Investigated influence of SARS-CoV infection on CXCL8 and CXCL10 in human intestinal epithelial cells. High concentrations of hydrocortisone prevented DNA binding activity of AP-1 and NF-kappabeta and inhibited upregulation of CXCL8 and CXCL10 but did not reduce chemokine expression to basal. Results suggest corticosteroid may be of limited benefit in suppression of chemokine production by SARS-CoV infected cells.
[34]	Plasma inflammatory cytokines and chemokines in severe acute respiratory syndrome.	Wong CK, Lam CW, Wu AK, Ip WK, Lee NL, Chan IH, Lit LC, Hui DS, Chan MH, Chung SS, Sung JJ	Hong Kong	2004	Overproduction of TNF-alpha, IL-1, IL-6 and IL-10 hallmark of viral infection. Investigated inflammatory profiles of patients infected with SARS in Hong Kong hospital. Use of corticosteroids significantly reduced IL-8, MCP-1 and IP-10 concentrations from 5-8 days after treatment.
[35]	Description and clinical treatment of an early outbreak of severe acute respiratory syndrome [18] in Guangzhou, PR China.	Zhao Z, Zhang F, Xu M, Huang K, Zhong W, Cai W, Yin Z, Huang S, Deng Z, Wei M, Xiong J, Hawkey PM	China	2003	Patients treated in different methods. 2 groups of 4 received steroids (in form of methyl prednisolone). Early use of high-dose steroids appeared to help recovery from SARS. Early use of high-dose steroids in combination with a quinolone plus azithromycin gave the best outcome with improvement of clinical signs and symptoms, decreased incidence of ARDs and mechanical ventilation as well as mortality. Hypothesised that steroids may help by reducing the damaging effect of the local inflammatory response.

**Supplementary Table 3.** Current studies investigating COVID-19/other coronavirus strains and TNF blockers. *(continued)*

Study Ref	Title	Authors	Country of study	Year	Summary of results
[36]	TNF-alpha inhibition for potential therapeutic modulation of SARS coronavirus infection.	Tobinick E.	US	2004	Clinical and experimental evidence implicate TNF as a possible mediator of the severe immune-based pulmonary injury which can follow infection with H5N1 influenza and SARS coronavirus. In humans, anti-TNF therapy utilising etanercept has been reported to be beneficial for treatment of the non-infectious idiopathic pneumonia syndrome which can follow stem-cell transplantation, a pulmonary syndrome that resembles SARS pneumonia in some respect. If the SARS coronavirus does indeed lead to massive release of TNF-αf rom alveolar macrophages, then early inhibition of TNF-α might be able to prevent TNF-αmediated immune activation and therefore reduce pulmonary injury in these patients. Compared with the use of corticosteroids, the use of biologic TNF inhibitors, including etanercept, has the potential to be a more specific and more effective method of ameliorating the severe alveolar damage which can occur following infection with these agents.
[37]	Anti-TNF alpha therapy does not ameliorate disease in a model of acute virus-endotoxin mediated respiratory disease in pigs.	Atanasova K; Van Gucht S; Van Reeth K.	Netherlands	2010	22 piglets were assessed to to elucidate the role of TNF-α in the development of virus-endotoxin-induced respiratory disease. The study demonstrates that after intratracheal administration etanercept is able to successfully block TNF-α activity in vivo in the lungs of PRCV-LPS inoculated pigs during the first 4–8 HPI. TNF-α reduction, however, was not associated with decrease in disease severity, bronchoalveolar neutrophil infiltration, or altered virus replication and induction of IL-1, IL-6, IL-12/IL-23 or IFN-α in the lungs. There was no obvious difference in macroscopic lung lesions and histopathological findings in the lungs. This data confirms the generally accepted belief that TNF-α, though very important, is not the sole culprit in development of respiratory disease and pathology, and possibly other, yet unidentified, components and mechanisms of the immune system are involved.

**Supplementary Table 4.** Current studies investigating COVID-19/ other coronavirus strains and IL-6. *(continued)*

Study Ref	Title	Authors	Country of study	Year	Summary of results
[38]	Diagnostic Utility of Clinical Laboratory Data Determinations for Patients with the Severe COVID-19.	Gao Y; Li T; Han M; Li X; Wu D; Xu Y; Zhu Y; Liu Y; Wang X; Wang L.	China	2020	They investigated forty‐three adult patients with COVID‐19. The patients were classified into mild group (28 patients) and severe group (15 patients). Comparison of the haematological parameters between the mild and severe groups showed significant differences in IL‐6, D‐Dimer, GLU, TT, FIB and CRP (*p* <0.05). Infection-related biomarkers appeared to differ between the two groups (IL-6).However, the proportion of IL-6 above normal was [36.10(23.00,59.20) pg/mL]in the severe group, which was significantly higher than that in the mild group [10.60(5.13,24.18) pg/mL]. The AUC of IL-6 which was used to predict the severity of COVID-19 was 0.795 (P<0.0001), which could better predict whetherrCOVID-19 was complicated by severe pneumonia. The optimum critical point of IL-6 in the group was 24.3 pg/ml, which was the upper limit of no severe pneumonia. IL‐6 and D‐Dimer were closely related to the occurrence of severe COVID‐19 in the adult patients, and their combined detection had the highest specificity and sensitivity for early prediction of the severity of COVID‐19 patients.
[39]	Induction of pro-inflammatory cytokines (IL-1 and IL-6) and lung inflammation by COVID-19: anti-inflammatory strategies.	Conti, P; Ronconi, G; Caraffa, A; Gallenga, C; Ross, R; Frydas, I; Kritas, S.	Italy	2020	When COVID-19 infect the upper and lower respiratory tract it can cause mild or highly acute respiratory syndrome with consequent release of pro-inflammatory cytokines, including interleukin (IL)-1b and IL-6. The binding of COVID-19 to the Toll Like Receptor (TLR) causes the release of pro-IL-1b which is cleaved by caspase-1, followed by inflammasome activation and production of active mature IL-1b which is a mediator of lung inflammation, fever and fibrosis. Proinflammatory cytokines levels are correlated with COVID-19 replication and disease. Suppression of pro-inflammatory IL-1 family members and IL-6 have been shown to have a therapeutic effect in many inflammatory diseases, including viral infections.
[40]	Inhibitory effects of glycopyrronium, formoterol, and budesonide on coronavirus HCoV-229E replication and cytokine production by primary cultures of human nasal and tracheal epithelial cells	Yamaya M; Nishimura H; Deng X; Sugawara M; Watanabe O; Nomura K; Shimotai Y; Momma H; Ichinose M; Kawase T.	Netherlands	2020	Primary human nasal (HNE) and tracheal (HTE) epithelial cell cultures were infected with HCoV-229E coronavirus. Pretreatment of HNE and HTE cells with glycopyrronium or formoterol decreased viral RNA levels and/or titers, the expression of the HCoV-229E receptor CD13, the number and fluorescence intensity of acidic endosomes where HCoV-229E RNA enters the cytoplasm, and the infection-induced production of cytokines, including IL-6, IL-8, and IFN-beta. IL-6 and IL-8 are related to airway inflammation in COPD and bronchial asthma exacerbation induced by viral infection. The decreased production of IL-6 and IL-8 in cells pretreated with glycopyrronium, formoterol, and budesonide, as well as the increased inhibitory effects of GFB observed in the present study, may be associated with the inhibitory effects of these drugs on COPD and bronchial asthma exacerbation.
[41]	Analysis of clinical features of 29 patients with 2019 novel **coronavirus** pneumonia	Chen, L; Liu, H G; Liu, W; Liu, J; Liu, K; Shang, J; Deng, Y; Wei, S.	China	2020	29 patients with 2019-ncov admitted to the isolation ward of Tongji hospital ( mild (15 cases), severe (9 cases) and critical (5 cases). The expression levels of inflammatory cytokines and other markers in the serum of each group were detected. There were statistically significant differences in the expression levels of interleukin-2 receptor (IL-2R) and IL-6** in the serum of the three groups (***p* < 0.05), among which the critical group was higher than the severe group and the severe group was higher than the mild group. However, there were no statistically significant differences in serum levels of tumor necrosis factor-alpha (TNF-alpha), IL-1, IL-8, IL-10, hs-CRP, lymphocyte count and LDH among the three groups (P>0.05).
[26]	Severe acute respiratory syndrome: clinical and laboratory manifestations.	Lam, Christopher W K; Chan, Michael H M; Wong, Chun K.	Australia	2004	They have investigated daily changes in plasma inflammatory cytokines and chemokines in 20 adult SARS patients [19 men and 1 woman, mean (SD) age 33 (12) years, range 21–58] for 19 consecutive days upon hospital admission (from ≤ 2 days after disease onset). The cytokine profile showed marked elevation of T-helper lymphocyte type 1 (Th1) cytokine IFN-γ, inflammatory cytokines IL-1β, IL-6 and IL-12 for at least two weeks after disease onset, but there was no significant increase in inflammatory cytokine TNF-α, anti-inflammatory cytokine IL-10, Th1 cytokine IL-2, and T-helper lymphocyte type 2 (Th2) cytokine IL-4. We have also performed serial studies of plasma cytokine and chemokine profiles of 8 children with SARS (5 boys and 3 girls, age 0.3 – 6.2 years) and found that these patients had a much milder cytokine and chemokine storm, rendering the use of corticosteroids more controversial if not unjustified.
[42]	Clinical Progression and Cytokine Profiles of Middle East Respiratory Syndrome Coronavirus Infection.	Kim ES; Choe PG; Park WB; Oh HS; Kim EJ; Nam EY; Na SH; Kim M; Song KH; Bang JH; Park SW; Kim HB; Kim NJ; Oh MD.	Korea (South)	2016	17 patients with laboratory-confirmed MERS-CoV during the 2015 outbreak in Korea were studied. The severe group had higher neutrophil counts during week 1 than the mild group (4,500 versus 2,200/muL, P = 0.026). In the second week of illness, the severe group had higher serum levels of IL-6 (54 versus 4 pg/ml, *p* = 0.006) and CXCL-10 (2,642 versus 382 pg/ml, *p* < 0.001).
[27]	Decoding the enigma of antiviral crisis: Does one target molecule regulate all?. [Review]	Mahmud-Al-Rafat A; Majumder A; Taufiqur Rahman KM; Mahedi Hasan AM; Didarul Islam KM; Taylor-Robinson AW; Billah MM	England	2019	IL-6 Interleukin acts as a mediator between pro- and anti-inflammatory reactivity by initiating trans- and classical-signalling, which might relate to the cytokine storm that is triggered by excessive pro-inflammatory responses to Ebola, SARS-CoV and dengue infections in humans. Future antivirals should thus aim to target the mechanism that regulates switching between IL-6 trans- and classical-signaling The tumour necrosis factor-α converting enzyme ADAM-17 could be the master molecule involved in regulating IL-6 class switching and through this in controlling pro- and anti-inflammatory responses to viral antigenic stimuli. ADAM-17 should be considered as a potential target molecule for novel antiviral drug discovery that would regulate host reactivity to infection and thereby limit or prevent fatal outcomes.
[43]	Extraordinary GU-rich single-strand RNA identified from SARS coronavirus contributes an excessive innate immune response.	Li Y; Chen M; Cao H; Zhu Y; Zheng J; Zhou H.	France	2013	They identified a set of SARS-CoV specific GU-rich ssRNA fragments with a high-density distribution in the genome. In vitro experiments, the result showed the representative SARS-CoV ssRNAs had powerful immunostimulatory activities to induce considerable level of pro-inflammatory cytokine TNF-a, IL-6 and IL-12 release via the TLR7 and TLR8, almost 2-fold higher than the strong stimulatory ssRNA40 that was found previously from other virus. Moreover, SARS-CoV ssRNA was able to cause acute lung injury in mice with a high mortality rate in vivo experiment. It suggests that SARS-CoV specific GU-rich ssRNA plays a very important role in the cytokine storm associated with a dysregulation of the innate immunity and could open a new therapeutic strategy.
[44]	Regulation of the p38 mitogen-activated protein kinase and dual-specificity phosphatase 1 feedback loop modulates the induction of interleukin 6 and 8 in cells infected with coronavirus infectious bronchitis virus.	Liao Y; Wang X; Huang M; Tam JP; Liu DX	US	2011	Characterised cellular mechanisms exploited by coronavirus infectious bronchitis virus (IBV) to regulate the induction of two pro-inflammatory cytokines, interleukin (IL)-6 and IL-8, at the transcriptional level. IBV modulates the infection by inducing the expression of dual-specificity phosphatase 1 (DUSP1), a negative regulator of the p38 MAPK, in order to limit the production of an excessive amount of IL-6 and IL-8 in the infected cells. DUSP1 and p38 MAPK are possible therapeutic targets for IBV.
[45]	A new mouse-adapted strain of SARS-CoV as a lethal model for evaluating antiviral agents in vitro and in vivo.	Day CW; Baric R; Cai SX; Frieman M; Kumaki Y; Morrey JD; Smee DF; Barnard DL.	US	2009	A new strain of SARS-CoV (strain v2163) was characterised and it was highly lethal in 5- to 6-week-old BALB/c mice. It had nine mutations affecting 10 amino acid residues. Strain v2163 increased IL-1alpha, IL-6, MIP-1alpha, MCP-1, and RANTES in mice, and high IL-6 expression correlated with mortality.
[46]	Neutralising antibody against severe acute respiratory syndrome -coronavirus spike is highly effective for the protection of mice in the murine SARS model.	Ishii K; Hasegawa H; Nagata N; Ami Y; Fukushi S; Taguchi F; Tsunetsugu-Yokota Y	Australia	2009	The efficacy of three SARS vaccine candidates was tested in a murine SARS model utilising low-virulence Pp and SARS-CoV coinfection. Vaccinated mice were protected from severe respiratory disease. A high level of IL-6 and on days 2 and 3 after SARS-CoV infection was closely linked to the virus replication and disease severity, suggesting the importance of these cytokines in the lung pathogenicity of SARS-CoV infection.
[47]	Severe acute respiratory syndrome (18) coronavirus-induced lung epithelial cytokines exacerbate SARS pathogenesis by modulating intrinsic functions of monocyte-derived macrophages and dendritic cells.	Yoshikawa T; Hill T; Li K; Peters CJ; Tseng CT.	US	2009	In highly polarised human lung epithelial Calu-3 cells modelled the cellular bases of the host antiviral innate immunity within the lungs. Role of IL-6 as a key SARS-CoV-induced epithelial cytokine capable of inhibiting the T-cell-priming ability of Denditric cells leading to an exacerbated inflammatory cascades and severe tissue damage in SARS patients.
[48]	Nucleocapsid protein of SARS-CoV activates interleukin-6 expression through cellular transcription factor NF-kappaB.	Zhang X; Wu K; Wang D; Yue X; Song D; Zhu Y; Wu J.	US	2007	High levels of IL6 High levels of interleukin-6 (IL-6) in the acute stage associated with lung lesions were found in SARS patients. The viral nucleocapsid SARS-CoV N protein** activate** IL-6 expression in the lung cells.
[49]	Cytokine regulation in SARS coronavirus infection compared to other respiratory virus infections.	Okabayashi T; Kariwa H; Yokota S; Iki S; Indoh T; Yokosawa N; Takashima I; Tsutsumi H; Fujii N.	US	2006	They compared the cytokine profile in Caco2 cells after infection of SARS coronavirus (SARS-CoV) with other respiratory viruses including respiratory syncytial virus (RSV), influenza A virus (FluAV), and human parainfluenza virus type 2 (hPIV2). Interferon (IFN) system (production and response) was not suppressed by SARS-CoV infection SARS-CoV and RSV induced high levels of IL-6 and RANTES compared with FluAV and hPIV2. Induction level of suppressor of cytokine signaling-3 (SOCS3) by SARS-CoV was significantly lower than that by RSV in spite of the significant production of IL-6. Collectively, overinduction of inflammatory cytokine and dysregulation of cytokine signaling may contribute to the immunopathology associated with “severe” inflammation in SARS.
[31]	A study of pulmonary inflammatory reaction induced by N-protein of SARS-CoV in rat models and effects of glucocorticoids on it	Hao D; He LX; Qu JM; Pan J; Hu BJ; Zhang J; Li ZZ.	China	2005	The pulmonary inflammatory reaction in rat models were induced by intratracheal instillation of N-protein of SARS-CoV and glucocorticoids were administrated to one of the groups. The N-protein of SARS-CoV presented pathogenicity and could induce pulmonary inflammatory reaction and acute lung injury, which were related to the increase and imbalance of pro-inflammatory and anti-inflammatory cytokines. Glucocorticoids could effectively alleviate the pulmonary inflammatory reaction induced by N-protein of SARS-CoV.
[50]	Clinical manifestations and inflammatory cytokine responses in patients with severe acute respiratory syndrome.	Sheng WH; Chiang BL; Chang SC; Ho HN; Wang JT; Chen YC; Hsiao CH; Hseuh PR; Chie WC; Yang PC.	Singapore	2005	Fourteen hospitalised patients with a diagnosis of SARS-associated coronavirus infection. There were no significant differences in peak levels of IL-6, IL-8 and TNF-alpha between patients with and without acute respiratory distress syndrome. CRP and TNF-alpha are associated with worse outcomes and might be used as prognostic markers of SARS.
[51]	Severe acute respiratory syndrome and the innate immune responses: modulation of effector cell function without productive infection.	Tseng CT; Perrone LA; Zhu H; Makino S; Peters CJ.	US	2005	SARS-CoV does not productively infect human macrophages or Dentritic cells, it appears to exert differential effects on Mphi and DC maturation and functions, which might contribute to SARS pathogenesis. It modulates a massive release of IL-6 and IL-12. However, the endocytic capacity (e.g., Ag capture) of Mphi was significantly compromised upon exposure to infectious SARS-CoV.
[52]	An interferon-gamma-related cytokine storm in SARS patients.	Huang KJ; Su IJ; Theron M; Wu YC; Lai SK; Liu CC; Lei HY.	US	2005	Fourteen cytokines or chemokines were analysed on 88 RT-PCR-confirmed severe acute respiratory syndrome [18] patients. IFN-gamma, IL-18, TGF-beta, IL-6, IP-10, MCP-1, MIG, and IL-8, but not of TNF-alpha, IL-2, IL-4, IL-10, IL-13, or TNFRI, were highly elevated in the acute phase sera of Taiwan SARS patients. IL-18, IP-10, MIG, and MCP-1 were significantly higher in the death group than in the survival group An interferon-gamma-related cytokine storm was induced post SARS coronavirus infection, and this cytokine storm might be involved in the immunopathological damage in SARS patients.
[53]	Temporal relationship of viral load, ribavirin, interleukin (IL)-6, IL-8, and clinical progression in patients with severe acute respiratory syndrome.	Wang WK; Chen SY; Liu IJ; Kao CL; Chen HL; Chiang BL; Wang JT; Sheng WH; Hsueh PR; Yang CF; Yang PC; Chang SC;	US	2004	8 patients with acute respiratory syndrome [18], were included to study the link between viral load, ribavirin, proinflammatory cytokines, and clinical progression. ribavirin was not effective in reducing the SARS coronavirus load in 3 of 8 probable cases studied elevated levels of interleukin (IL)-6 and IL-8 subsequent to the peak viral load were found in 8 and 6 cases, respectively.
[54]	Analysis of serum cytokines in patients with severe acute respiratory syndrome.	Zhang Y; Li J; Zhan Y; Wu L; Yu X; Zhang W; Ye L; Xu S; Sun R; Wang Y; Lou J.	US	2004	Serum from 110 individuals (healthy donors, patients with SARS, patients with severe SARS, and patients with SARS in convalescence) was collected and cytokine profile was studied. The IL-6 concentration was increased in SARS patients and was significantly elevated in severe SARS patients, but the IL-6 concentrations were similar in convalescent patients and control subjects, suggesting that there was a positive relationship between the serum IL-6 concentration and SARS severity. The concentrations of IL-8 and TGF-beta were decreased in SARS patients and significantly reduced in severe SARS patients, but they were comparable in convalescent SARS patients and control subjects, suggesting that there was a negative relationship between the IL-8 and TGF-beta concentrations and SARS severity. The concentrations of IL-1 and TNF-alpha were not significantly different in different groups.
[34]	Plasma inflammatory cytokines and chemokines in severe acute respiratory syndrome.	Wong CK; Lam CW; Wu AK; Ip WK; Lee NL; Chan IH; Lit LC; Hui DS; Chan MH; Chung SS; Sung JJ.	England	2004	Changes in plasma T helper (Th) cell cytokines, inflammatory cytokines and chemokines in 20 patients diagnosed with SARS were assessed. Th1 cytokine interferon (IFN)-gamma, inflammatory cytokines interleukin (IL)-1, IL-6 and IL-12 was elevated for at least 2 weeks after disease onset, but there was no significant elevation of inflammatory cytokine tumour necrosis factor (TNF)-alpha, anti-inflammatory cytokine IL-10, Th1 cytokine IL-2 and Th2 cytokine IL-4. The chemokine profile demonstrated significant elevation of neutrophil chemokine IL-8, monocyte chemoattractant protein-1 (MCP-1), and Th1 chemokine IFN-gamma-inducible protein-10 (IP-10). Corticosteroid reduced significantly IL-8, MCP-1 and IP-10 concentrations from 5 to 8 days after treatment (all p < 0.001).
[55]	Dynamic changes and the meanings of blood cytokines in severe acute respiratory syndrome	Wang and Pang	China	2003	(Abstract only) This study observed changed in various serum IL levels in patients with SARS. The authors stated that the mean concentration of serum IL-6 in SARS patients did not differ from the control group in 3-7-day group and 8-14-day group, but became significantly higher in over 14-day group as compared to the control group, 3-7-day group and 8-14-day group (*p* < 0.01). The results of the study led the authors to conclude that the immune state of SARS was abnormal. However, as only the abstract was available it is hard to draw upon any more conclusions from this study in relation in IL-6 specifically.
[56]	Inflammatory Cytokine Profile in Children With Severe Acute Respiratory Syndrome	Ng *et al*	Hong Kong	2004	The authors of this study set out to study the inflammatory cytokine profile in children with SARS. They found that the plasma concentrations key proinflammatory cytokines, including IL-6 were not substantially increased in any of the patients throughout the course of illness. From this the authors stated that the cytokine results cast doubt on the liberal use of corticosteroids in paediatric SARS patients, as the host immunologic response did not seem to be as severe as initially anticipated.

**Supplementary Table 5.** Current studies investigating COVID-19/ other coronavirus strains and JAK inhibitors

Study Ref	Title	Authors	Country of study	Year	Summary of results
[57]	Baricitinib as potential treatment for 2019-nCoV acute respiratory disease	Richardson *et al*	England	2020	ACE2 is a cell-surface protein on lung cells in corona viral infected patients AAK1 receptor promoted endocytosis involved in ACE2 The study suggests the use of Baricitinib to inhibit AAK1 in patients with 2019-nCoV acute respiratory disease, to reduce both the viral entry and the inflammation in patients, using endpoints such as the MuLBSTA score, an early warning model for predicting mortality in viral pneumonia [58].
[59]	The Coronavirus Transmissible Gastroenteritis Virus Evades the Type I Interferon Response through IRE1α-Mediated Manipulation of the MicroRNA miR-30a-5p/SOCS1/3 Axis	Ma *et al*	China	2018	JAK-signal transducer and activator of transcription (STAT), the suppressor of cytokine signaling protein 1 (SOCS1), and SOCS3. IFN-I are major antiviral molecules, and coronaviruses have evolved diverse strategies to counter the IFN-I response during infection. This study uses endoplasmic reticulum stress and IFN-I production after infection with transmissible gastroenteritis virus (TGEV) to understand how coronavirus-elicited ER stress is actively involved in viral replication and manipulates the host IFN-I response. Increased SOCS1 or SOCS3 expression impaired the IFN-I antiviral response, promoting TGEV replication. IRE1α is an endoplasmic reticulum stress sensor, which led to the increased expression of negative regulators of JAK-STAT SOCS1 and SOCS3. Therefore, IRE1α may be a novel target against coronavirus infection.
[60]	Targeting Coronaviral Replication and Cellular JAK2 Mediated Dominant NF-kappaB Activation for Comprehensive and Ultimate Inhibition of Coronaviral Activity	Yang *et al*	Taiwan	2017	Tylophorine-based compounds exert broad spectral, potent inhibition of coronaviruses. NF-**κB** activation is a common pro-inflammatory response of host cells to viral infection. The combination treatment, wherein a tylophorine compound targets TGEV and a JAK2 inhibitor blocks the alternative dominant NF-**κB** activation mediated by JAK2, is more effective and comprehensive than either one alone and constitutes a feasible approach for the treatment of SARS-CoV or MERS-CoV
[61]	Severe Acute Respiratory Syndrome Coronavirus Evades Antiviral Signaling: Role of nsp1 and Rational Design of an Attenuated Strain	Wathelet *et al*	USA	2007	Expression of non-structural protein 1 (nsp1) significantly inhibited the activation of SARS-Cov signaling pathways. However, the study results show that SARS-CoV nsp1 does not inhibit JAK phosphorylation.

**Supplementary Table 6.** Current studies investigating COVID-19/ other coronavirus strains and IL-1 blockade.

Study Ref	Title	Authors	Country of study	Year	Summary of results
[62]	Analysis of clinical features of 29 patients with 2019 novel coronavirus pneumonia.	Chen *et al*	China	2020	The levels of several inflammatory markers were measured in 29 COVID-19 patients and were compared between general, severe and critically ill groups. The authors reported no significant differences in IL-1b levels between the three groups of patients.
[63]	Rat coronaviruses infect rat alveolar type I epithelial cells and induce expression of CXC chemokines.	Miura *et al*	USA	2007	The authors used two rat coronaviruses on alveolar epithelial cells taken from 6-8 weeks old rats. Infection with the viruses caused the increase in expressed of both IL-1a and IL-1b. The authors concluded that the virus-induced chemokine expression was subsequently reduced by the IL-1 receptor antagonist, suggesting that IL-1 produced by infected cells induces uninfected cells to express chemokines.
[64]	Role of the inflammasome-related cytokines Il-1 and Il-18 during infection with murine coronavirus.	Zalinger, Elliott and Weiss	USA	2017	The authors used several infectious agents including murine coronavirus to assess the role of the inflammasome and its related cytokines on pathogenesis and host defence during viral infection. The authors concluded that mice lacking IL-1 signalling experienced elevated viral replication but similar survival compared to wild-type controls.
[65]	Severe Acute Respiratory Syndrome Coronavirus Viroporin 3a Activates the NLRP3 Inflammasome.	Chen *et al*	Japan and Taiwan	2019	The authors provide evidence that SARS-CoV 3a protein activates the NLRP3 inflammasome in lipopolysaccharide-primed macrophages and that the ion channel activity of the 3a protein was essential for 3a-mediated IL-1β secretion. The macrophages obtained in this study were from 6-week-old female mice.
[66]	Mast cells contribute to coronavirus-induced inflammation: new anti-inflammatory strategy.	Kritas *et al*	Greece, Italy and USA	2020	(Abstract Only) Infection with coronavirus activates mast cells which in turn causes the generation of pro-inflammatory IL-1 family members. The authors state that they demonstrate for the first time that inflammation by coronavirus may be inhibited by anti-inflammatory cytokines belonging to the IL-1 family. However, as this is only an abstract there is no further information to support this statement.
[67]	SARS Immunity and Vaccination.	Zhu M	China	2004	SARS-CoV binds to host cells via a specific SARS receptor, angiotensin converting enzyme 2 (ACE-2). Following entry into the cell, the virus uncoats, nucleic acid is released and transcription occurs for production of viral proteins. During this course, the host immune system is activated (B and T-cells). Specifically, CD4+ T helper cells recognise antigenic peptides produced by antigen presenting cells and produce cytokines that promote cell mediated and/or humoral immunity. Similarly to avian flu, SARS infection induced similar pro-inflammatory cytokine pattern and might contribute to the severe nature of the virus. Clinical evidence of SARS treatment by corticosteroids where levels of IL-1beta reduced after administration- thus inhibition of inflammatory cytokines such as IL-1 may be beneficial strategy for treatment of SARS.
[54]	Analysis of serum cytokines in patients with severe acute respiratory syndrome.	Zhang Y, Li J, Zhan Y, Wu L, Yu X, Zhang W, Ye L, Xu S, Sun R, Wang Y, Lou J	China	2004	Following the discovery of SARS-CoV, no specific or efficient clinical treatments were available since pathogenesis was not well understood. Acute lung injury associated with SARS can be attributed to complex and multifactorial pathophysiological process involving cytokines. Demonstrated that IL-1 play key role in pathogenesis of acute lung injury. WHO inferred that severe immune response kills SARS patients and that some cytokines may play important role in the process. IL-1 cytokine promotes inflammation by inducing cell injury. Study measured levels of IL-1 in 4 patient groups: controls, patients with SARS, patients with severe SARS and convalescent SARS patients. Study showed that levels of IL-1alpha did not differ between 3 SARS groups or controls. Suggests that host immune response to novel coronavirus may be different from the immune reaction to other pathogens. Overall results suggest that acute lung injury associated with SARS may not be induced by circulating cytokines. HOWEVER some patients had accepted corticosteroids and therefore results consistent with reports that only long term treatment (7-10 days) with a steroid can alter serum cytokine levels.
[34]	Plasma inflammatory cytokines and chemokines in severe acute respiratory syndrome.	Wong CK, Lam CW, Wu AK, Ip WK, Lee NL, Chan IH, Lit LC, Hui DS, Chan MH, Chung SS, Sung JJ	Hong Kong	2004	Known that over-expression of IL-1beta hallmark of SARS infection probably through activation of transcription factor nuclear factor, activator protein AP1 and activating factor 2. Therefore lung damage associated with SARS postulated to occur through cytokine and chemokine dysregulation. Study involved measurement of cytokines and chemokines including IL-1beta in 20 consecutive patients admitted to Hong Kong hospital. IL-1beta was significantly elevated above normal range within first 12, 7 and 5 days after disease onset. Those patients with more severe disease treated with pulsed methylprednisolone. IL-1beta levels higher in this patient group particularly 3-10 days after onset but returned to normal levels 1 week after disease onset in both sets of patients. IL-1beta can act as early response cytokine to viral infection. IL-1 elevation in SARS can induce hyperinnate inflammatory response leading to recruitment and accumulation of alveolar macrophages and PMN. Higher disease severity associated with more elevated plasma IL-1beta and addition of pulsed steroid controlled rapidly deteriorating clinical condition and attenuated otherwise exaggerated immunological response.

**Supplementary Table 7.** Current studies investigating COVID-19/ other coronavirus strains and Mycophenolate.

Study Ref	Title	Authors	Country of study	Year	Summary of results
[68]	Interferon-β and mycophenolic acid are potent inhibitors of middle east respiratory syndrome coronavirus in cell-based assays	Hart *et al*	USA	2014	(*In vitro* only) MPA showed strong inhibition, with an IC50 of 2.87 mM. This drug may provide an alternative to ribavirin for treatment of MERS-CoV. In conclusion, IFN-b, MPA or a combination of the two may be beneficial in the treatment of MERS-CoV or as a post-exposure intervention in high-risk patients with known exposures to MERS-CoV.
[69]	Disulfiram can inhibit MERS and SARS coronavirus papain-like proteases via different modes	Lin *et al*	Taiwan	2018	(In vitro) Note. (PLpros)= Papain-like proteases MERS-CoV and SARS-CoV – act as a potential anti-viral target for drugs Multiple inhibition assays also support a kinetic mechanism by which disulfiram together with 6TG and/or MPA can synergistically inhibit MERS-CoV PLpro, but not, due to its competitive mode of inhibition, SARS-CoV PLpro. Synergistic inhibition of MERS-CoV PLpro by disulfiram and 6-thioguanine or **mycophenolic acid** implies the potential for combination treatments using these three clinically available drugs.
[70]	Thiopurine analogs and mycophenolic acid synergistically inhibit the papain-like protease of Middle East respiratory syndrome coronavirus	Cheng et al	Taiwan	2015	(In vitro) At the time of the study, there were no potent inhibitors that target MERS-CoV PLpro. The authors report that the immunosuppressive drug mycophenolic acid, was able to inhibit MERS-CoV PLpro. MPA is a non-competitive inhibitor of MERS-CoV PLpro.
[71]	Broad-spectrum antivirals for the emerging Middle East respiratory syndrome coronavirus	Chan *et al*	Hong Kong/China	2013	(*In vitro*) The authors assessed the anti-MERS-CoV activities of several compounds. Only mycophenolic acid exhibited low EC50 and high selectivity index. They found that a combination of mycophenolic acid and IFN-b1b lowered the EC50 of each drug by 1-3 times. Mycophenolic acid exhibits a number of attributes that support its practical use in MERS-CoV infection. It is commonly available in two forms, the prodrug mycophenolate mofetil and the salt mycophenolate sodium, and could be given orally. Interferon-b1b with mycophenolic acid should be considered in treatment trials of MERS.
[72]	High-Throughput Screening and Identification of Potent Broad-Spectrum Inhibitors of Coronaviruses.	Shen *et al*	China	2019	*(In vitro)* The authors sought to identify potent broad-spectrum inhibitors of corona viruses. They concluded that they had identified seven compounds (lycorine, emetine, monensin sodium, **mycophenolate mofetil, mycophenolic acid**, phenazopyridine, and pyrvinium pamoate) as broad-spectrum inhibitors according to their strong inhibition of replication by four CoVs in vitro at low-micromolar concentrations. They all inhibited the replication of all CoVs with EC50 values of 5μm. Mycophenolate mofetil and mycophenolic acid, showed a similar antiviral effect on the four CoVs suggesting that the two drugs might harbour similar core structures and antiviral mechanisms. In vivo antiviral activity is unknown from this study.
[73]	Glycyrrhizin, an active component of liquorice roots, and replication of SARS-associated coronavirus	Cinatl *et al*	Germany	2003	(In vitro but using clinical samples - Abstract only) The authors assessed the antiviral activities of ribavirin, 6-azauridine, pyrazofurin, **mycophenolic acid**, and glycyrrhizin against two clinical isolates of coronavirus (FFM-1 and FFM-2) from patients with SARS admitted to the clinical centre of Frankfurt University, Germany. Mycophenolic acid did not affect replication of the SARS-associated coronaviruses.
[74]	Treatment outcomes for patients with Middle Eastern Respiratory Syndrome Coronavirus (MERS CoV) infection at a coronavirus referral center in the Kingdom of Saudi Arabia.	Al Ghamdi et al	Saudi Arabia	2016	(Clinical example) Eight patients (15.7%) received mycophenolate mofetil, seven of these patients received it in combination with interferon beta. All patients who received mycophenolate mofetil survived. The authors concluded that whilst treatment with beta interferon and mycophenolate mofetil predicted survival (in univariate analysis only), the greatest predictor of survival was the severity of illness on presentation.
[75]	Treatment with lopinavir/ritonavir or interferon-beta1b improves outcome of MERSCoV infection in a nonhuman primate model of common marmoset	Chan *et al*	Hong Kong/China	2015	(In vivo) The authors assessed 3 repurposed drugs with potent in vitro anti-MERS-CoV activity (mycophenolate mofetil, lopinavir/ritonavir, and interferon-β1b) in common marmosets with severe disease resembling MERS in humans. all MMF-treated animals developed severe and/or fatal disease with higher mean viral loads. The authors concluded that MMF alone may worsen MERS and should not be used.
[76]	Enhancement of the infectivity of SARS-CoV in BALB/c mice by IMP dehydrogenase inhibitors, including ribavirin	Barnard *et al*	USA	2006	In vivo study in BALB/c mice (a replication model for SARS infections) In mycophenolic acid-treated mice, the lung virus titers (aka viral load) increased with increasing dosage, although these increases in lung virus titers were not quite significantly different from the lung virus titers in placebo-treated, infected mice.
[77]	A review of treatment modalities for Middle East Respiratory Syndrome	Mo and Fisher	Singapore	2016	(Review) Mycophenolic acid also inhibits purine nucleotide synthesis in lymphocytes. This makes it a popular immunosuppressant in solid-organ transplants and autoimmune diseases such as systemic lupus erythematosus. The use of mycophenolic acid monotherapy has not been reported in MERS. IFN-b and mycophenolic acid combination therapy was described in a retrospective observational study in Saudi Arabia involving 51 patients; all of the 8 patients who received IFN-b and mycophenolic acid survived. Corticosteroids, ribavirin monotherapy and mycophenolic acid are likely to cause more harm than benefit. There are no clinical data on the efficacy of mycophenolic acid in SARS or MERS. However, it led to severe and/or fatal disease with higher mean viral loads in an animal model.
[78]	Update on therapeutic options for Middle East Respiratory Syndrome Coronavirus (MERS-CoV)	Al-Tawfiq and Memish	Saudi Arabia	2017	(Review) MPA mechanism of action - Direct and indirect antiviral activity by modulation of IFN response The use of mycophenolate in the common marmoset animal model resulted in higher mortality than untreated animals: Mortality rate was 67% (untreated and MMF treated) at 36 h post inoculation versus 0%–33% (lopinavir/ ritonavir-treated and interferon-β1b- treated
[79]	Middle East Respiratory syndrome coronavirus: Another zoonotic betacoronavirus causing SARS-like disease	Chan et al	Hong Kong/China	2015	(Review) The combination of mycophenolic acid and interferon beta 1b shows synergistic activity against MERS-CoV in Vero cells. The desirable pharmacokinetics of mycophenolic acid compared to ribavirin warrants further evaluation, although the potential inhibitory effect on the immune system and therefore neutralising antibody production should be fully assessed in animal models before use in humans. A fatal case of MERS was reported in a renal transplant recipient who was receiving antirejection therapy consisting of prednisone, mycophenolate mofetil, and cyclosporine, but the dosage, serum drug level of mycophenolate mofetil, and resulting lymphocyte count were not reported
[80]	A review of candidate therapies for Middle East respiratory syndrome from a molecular perspective.	Rabaan *et al*	Saudi Arabia	2017	(Review) Reiteration of the promising results of MPA in an in vitro setting, however, the marmoset animal model resulted in fatal disease and high viral loads. The authors therefore state that MPA should be used with caution for treatment of MERS-CoV.

The EC50 is the concentration of a drug that gives half-maximal response. The IC50 is the concentration of an inhibitor where the response (or binding) is reduced by half. These terms are referred to a few times within the summary table.

**Supplementary Table 8.** Current studies investigating COVID-19/ other coronavirus strains and Tacrolimus.

Study Ref	Title	Authors	Country of study	Year	Summary of results
[81]	Replication of human coronaviruses SARS-CoV, HCoV-NL63 and HCoV-229E is inhibited by the drug FK506	Carbajo-Lozoya *et al*.	Germany/Switzerland	2012	Coronaviruses represent the group of RNA viruses with the largest RNA genome to date therefore the development of resistant mutants to targeted drugs remains a concern. Viral replication represents potential antiviral targets. Study performed genome-wide SARS-CoV yeast-two-hybrid interaction screen with human cDNA libraries identifying FK506-binding proteins as interaction partners of SARS-CoV non-structural protein 1 (Nsp1). FK506-binding proteins bind the immunosuppressive drug FK506 (tacrolimus). Since Nsp1 interacts with FK506-binding proteins, investigated whether FK506 inhibits replication of human coronaviruses. VeroFM cells infected with SARS-CoV and other human coronaviruses and treated with FK506. Study found that FK506 inhibits the replication of SARS-CoV, HCoV-NL63 and HCoV-229E at non-toxic, low-micromolar concentrations with reduction in viral titers to undetectable levels. All human coronaviruses sensitive to FK506 indicating the involvement of FK506-binding proteins in viral replication.
[82]	MERS CoV infection in two renal transplant recipients: Case report	Al Ghamdi et al	Saudi Arabia	2015	Pneumonia caused by MERS-CoV associated with severe morbidity and mortality in immunocompromised patients. Data on clinical picture in solid organ transplant recipients and effect of anti-rejection immunosuppressive regimens unclear. 30-year old patient with renal transplant on immunosuppressive regimen of tacrolimus. Tested positive for MERS CoV through nasopharyngeal swab. Received antibacterial therapy and mycophenolate mofetil dose reduced and underwent full recovery. Difficult to pinpoint why made full recovery compared to other case study (not on tacrolimus) who died.
[83]	Human coronavirus NL63 replication is cyclophilin A-dependent and inhibited by non-immunosuppressive cyclosporine A-derivatives including Alisporivir	Carbajo-Lozoya *et al*	Germany	2014	Previous study shown that FK506 inhibit coronavirus replication. This study further shows that novel non-immunosuppressive derivatives of CsA and FK506 strongly inhibit the growth of human coronavirus HCoV-NL63 at low-micromolar, non-cytotoxic concentrations in cell culture. HCoV-NL63 and HCoV-HKU1 discovered in 2004 and 2005 causing more severe lower respiratory tract infections in younger children. PPIase-independent activities of CsA and FK506 exerted by gain-of-function, result from binary complexes formed by binding of the drugs to FKBPs. Based on inhibition of the protein phosphatase activity of calcineurin, these complexes block the cellular calcineurin/NFAT pathway thereby interfering with T-cell activation and IL-2 production. FK506 analogues (altered by side chain modification)

**Table 1.** Search terms and the number of studies included for each investigated drug group.

Drug group	Search terms	Number of studies included
All cytotoxic chemotherapy	(SARS-CoV-2 or coronavirus or COVID or betacoronavirus or Middle East Respiratory Syndrome Coronavirus (MERS-CoV) or SARS-CoV AND chemotherapy)	24/30
Low-dose steroids/NSAIDs	(SARS-CoV-2 or coronavirus or COVID or betacoronavirus or MERS-CoV or SARS-CoV) AND (anti-inflammatory or ibuprofen or isobutylphenylpropionic acid or cortisone or non-steroidal anti-inflammatory)	13/58
Any TNF blocker	((SARS-CoV-2 or coronavirus or COVID or betacoronavirus or MERS-CoV or SARS-CoV) AND (TNF blocker or Anti-TNF therapy or TNFα inhibitor or infliximab or etanercept or Certolizumab or Golimumab or adalimumab))	2/3
IL-6 blockade	Two different search strategies were explored due to the number of agents that block the IL-6: ((SARS-CoV-2 or coronavirus or COVID or betacoronavirus or MERS-CoV or SARS-CoV) AND (Anti-IL-6 therapy or anti-interleukin-6 receptor antibody or anti-interlukin-6 therapy or interlukin 6 blockage or interlukin-6 blockage or IL 6 blockage or IL-6 blockage or tocilizumab or siltuximab or Sylvant or sarilumab or olokizumab or CDP6038 or elsilimomab or BMS-945429 or ALD518 or sirukumab or CNTO 136 or CPSI-2364 or ALX-0061 or clazakizumab or olokizumab or sarilumab or sirukumab or ARGX-109 or FE301 or FM101)) ((SARS-CoV-2 or coronavirus or COVID or betacoronavirus or MERS-CoV or SARS-CoV) and (IL-6 or interlukin 6 or Anti-IL-6 therapy or IL-6 blockage or tocilizumab or siltuximab))	0 23/108
JAK inhibitors	((SARS-CoV-2 or coronavirus or COVID or betacoronavirus or MERS-CoV or SARS-CoV) and (JAK or JAK1 or JAK2 or TYK3 or tofacitinib or baricitinib or filgotinib or peficitinib or ABT494 or decernotinib))	4/15
IL-1 blockade	(SARS-CoV-2 or coronavirus or COVID or betacoronavirus or MERS-CoV or SARS-CoV) AND (Interleukin-1 or IL-1 or IL-1RA or canakinumab or anti-IL-1 or IL-1 antagonists or IL-1 blockers or rilonacept or IL-1 trap or ACZ885 or anakinra)	9/37
Mycophenylate	(SARS-CoV-2 or coronavirus or COVID or betacoronavirus or MERS-CoV or SARS-CoV) AND (mycophenolate mofetil OR mycophenolate OR myfortic)	13/29
Tacrolimus	(SARS-CoV-2 or coronavirus or COVID or betacoronavirus or MERS-CoV or SARS-CoV) AND (envarsus or tacni or tacrolimus or prograf or FK506)	3/18
Anti-CD20	((SARS-CoV-2 or coronavirus or COVID or betacoronavirus or MERS-CoV or SARS-CoV) AND (anti-cd20 monoclonal antibodies or anti-cd20 or rituximab or truxima or zevalin or ruxience or rituxan or arzerra or gazyva)	0
CTLA4-Ig	((SARS-CoV-2 or coronavirus or COVID or betacoronavirus or MERS-CoV or SARS-CoV) AND (CTLA-4 Ig or CTLA4 or CTLA-4 or Ipilimumab or yervoy)	0
